# Predicting DWI-FLAIR mismatch on NCCT: the role of artificial intelligence in hyperacute decision making

**DOI:** 10.3389/fneur.2023.1201223

**Published:** 2023-06-12

**Authors:** Beom Joon Kim, Kairan Zhu, Wu Qiu, Nishita Singh, Rosalie McDonough, Petra Cimflova, Fouzi Bala, Jongwook Kim, Yong Soo Kim, Hee-Joon Bae, Bijoy K. Menon

**Affiliations:** ^1^Department of Neurology, Seoul National University Bundang Hospital, Seongnam-si, Republic of Korea; ^2^Gyeonggi Regional Cerebrovascular Center, Seoul National University Bundang Hospital, Seongnam-si, Republic of Korea; ^3^College of Electronic Engineering, Xi’an Shiyou University, Xi’an, Shaanxi, China; ^4^School of Life Science and Technology, Huazhong University of Science and Technology, Wuhan, Hubei, China; ^5^Department of Clinical Neurosciences and Diagnostic Imaging, University of Calgary Cumming School of Medicine, Calgary, AB, Canada; ^6^Neurology Division, Department of Internal Medicine, Max Rady College of Medicine, University of Manitoba, Winnipeg, MB, Canada; ^7^Department of Medical Imaging, St Anne's University Hospital Brno and Faculty of Medicine, Masaryk University, Brno, Czechia; ^8^Diagnostic and Interventional Neuroradiology Department, University Hospital of Tours, Tours, France; ^9^Department of Neurology, Nowon Eulji Medical Center, Eulji University, Seoul, Republic of Korea; ^10^Department of Neurology, College of Medicine, Seoul National University, Seoul, Republic of Korea

**Keywords:** artificial intelligence, DWI, FLAIR, DWI-FLAIR mismatch, non-contrast computed tomography

## Abstract

**Background:**

The presence of diffusion-weighted imaging (DWI) and fluid-attenuated inversion recovery (FLAIR) mismatch was used to determine eligibility for intravenous thrombolysis in clinical trials. However, due to the restricted availability of MRI and the ambiguity of image assessment, it is not widely implemented in clinical practice.

**Methods:**

A total of 222 acute ischemic stroke patients underwent non-contrast computed tomography (NCCT), DWI, and FLAIR within 1 h of one another. Human experts manually segmented ischemic lesions on DWI and FLAIR images and independently graded the presence of DWI-FLAIR mismatch. Deep learning (DL) models based on the nnU-net architecture were developed to predict ischemic lesions visible on DWI and FLAIR images using NCCT images. Inexperienced neurologists evaluated the DWI-FLAIR mismatch on NCCT images without and with the model’s results.

**Results:**

The mean age of included subjects was 71.8 ± 12.8  years, 123 (55%) were male, and the baseline NIHSS score was a median of 11 [IQR, 6–18]. All images were taken in the following order: NCCT – DWI – FLAIR, starting after a median of 139 [81–326] min after the time of the last known well. Intravenous thrombolysis was administered in 120 patients (54%) after NCCT. The DL model’s prediction on NCCT images revealed a Dice coefficient and volume correlation of 39.1% and 0.76 for DWI lesions and 18.9% and 0.61 for FLAIR lesions. In the subgroup with 15  mL or greater lesion volume, the evaluation of DWI-FLAIR mismatch from NCCT by inexperienced neurologists improved in accuracy (from 0.537 to 0.610) and AUC-ROC (from 0.493 to 0.613).

**Conclusion:**

The DWI-FLAIR mismatch may be reckoned using NCCT images through advanced artificial intelligence techniques.

## 1. Introduction

The decision for intravenous thrombolysis administration strongly depends on the time elapsed since the onset of the stroke (1, 2). The time-based decision is based on findings from a group modeling study that demonstrated diminishing survival of ischemic penumbra and recanalization therapy benefits with increasing time since onset (3, 4). However, individual variation in infarct progression is likely a result of a balance between collateral status, the frailty of brain tissue, and the severity of the ischemic injury (5–7).

In lieu of the time from onset, which may be an inadequate surrogate for tissue ischemia, the mismatched lesion on the diffusion-weighted imaging (DWI) and fluid-attenuated inversion recovery (FLAIR) image has been proposed as a means of measuring the extent of individual ischemic brain injury (8, 9). Due to the fact that DWI images were more sensitive to early ischemic changes than FLAIR images, the phrase “tissue clock” was coined to describe its potential to use as criteria for thrombolysis (10). Randomized clinical trials that chose intravenous thrombolysis candidates in unclear onset time based on the diffusion-FLAIR mismatch demonstrated the feasibility and applicability of the tissue clock in acute decision-making (11–13).

However, the DWI-FLAIR mismatch is not commonly accepted in acute stroke care. The mismatch necessitates magnetic resonance image (MRI) scans, which have limited accessibility in acute stroke care around the world (14). Instead, computed tomography (CT) is the *de facto* standard for neuroimaging of acute stroke. Nevertheless, non-contrast CT (NCCT) has inferior spatial resolution and signal differentiation in comparison to MRI (15). Therefore, no report has tried to evaluate the DWI-FLAIR mismatch on NCCT scans of acute ischemic stroke suspects. With the development of artificial intelligence (AI) modeling for medical imaging, it is possible to create an AI model with automated image interpretation capability equivalent to that of human experts (16). In this study, the authors analyzed data from patients presenting with acute ischemic stroke who completed NCCT and MR images within an hour to build a deep-learning model that could predict the DWI-FLAIR mismatch using NCCT. The performance of this model was then compared to inexperienced human raters.

## 2. Materials and methods

### 2.1. Study participants

**Figure 1:**
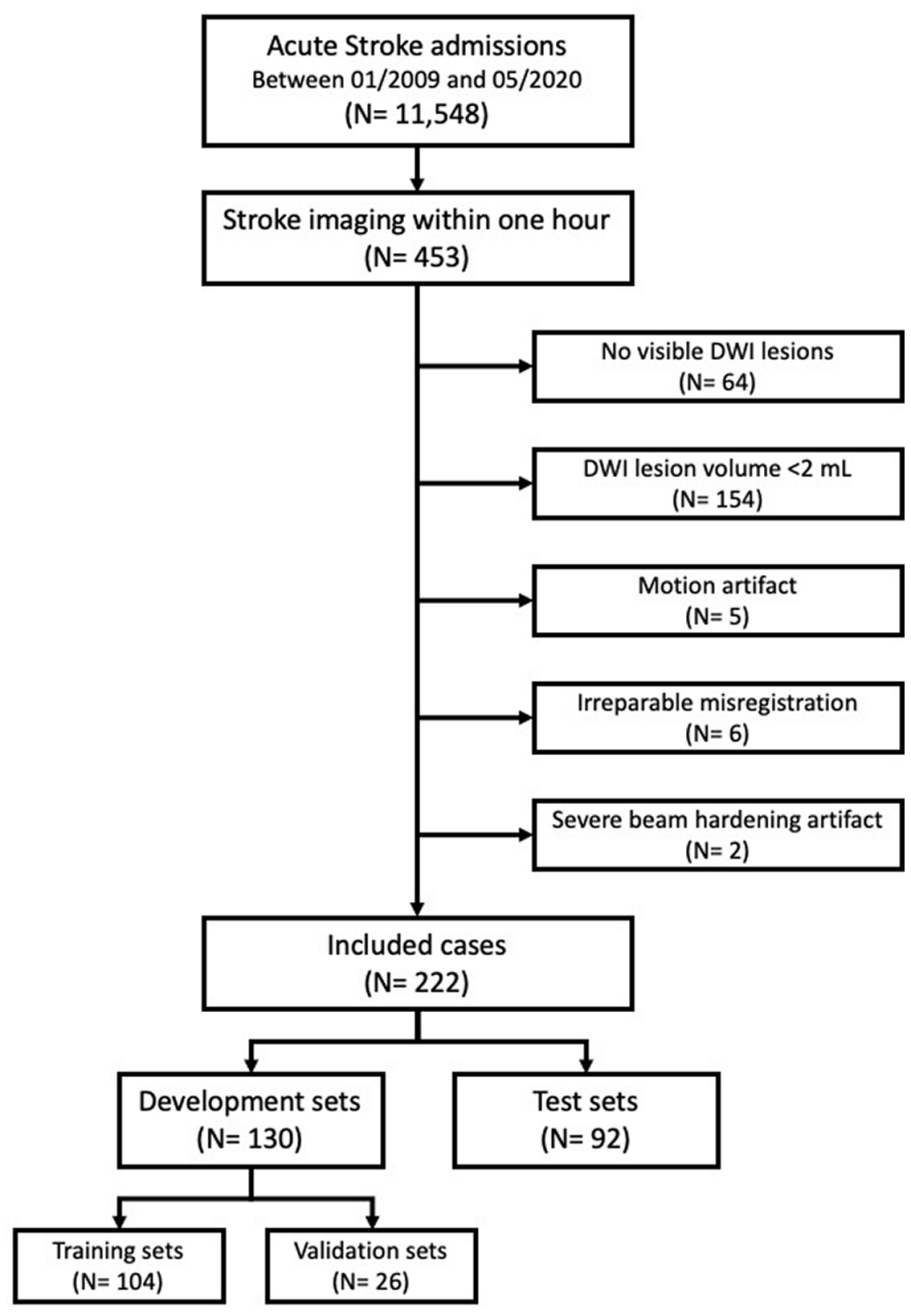
Study profile.

Between 01/2009 and 05/2020, a total of 11,548 consecutive acute stroke patients were enrolled in a prospective stroke registry at a single academic center (17). A total 453 patients were selected for this study who completed NCCT, DWI, and FLAIR image scans within 1 h of each other by the acquisition time (DICOM tag; 0008, 0032). Excluded patients were those with (1) no DWI lesion (*n* = 64); (2) DWI lesion volume smaller than 2 mL (*n* = 154); (3) significant motion artifacts (*n* = 5), irreparable misregistration (*n* = 6) and severe beam hardening artifacts (*n* = 2). The final analysis included 222 image sets of NCCT, DWI, and FLAIR images. Further, 130 image sets (104 training and 26 validation) were randomly assigned to the development set and the remaining 92 image sets were assigned to the test set (Figure 1).

Acute stroke management, including baseline and follow-up imaging, was undertaken in accordance with the current clinical guidelines at the time of care and at the discretion of treating physicians (1, 2, 18). The institutional stroke care protocol recommends taking CT images (includes NCCT, multiphase CT angiography, and CT perfusion) for patients suspected of having an acute stroke. If no contraindications, intravenous thrombolysis was administered after NCCT. Following CT, patients undergo acute MR scans (including DWI, FLAIR, MRA angiography, and MR perfusion) prior to admission to a stroke unit. If deemed suitable for endovascular therapy, the patient could directly proceed to the angiography suite following CT or MR scans.

Clinical information was retrieved from the prospective acute stroke registry. The institutional review board of the Seoul National University Bundang Hospital approved the data analysis, image evaluation, and modeling process (B-2102/667-106). Included patients or their next of kin provided written consent for the prospective clinical stroke registry to record and for the collection of their data (B-1401/236-007, B-1706/403-303).

### 2.2. Data availability statement

The data supporting this study’s findings are available from the corresponding author upon reasonable request.

### 2.3. Image acquisition

All patients had their NCCT scans using 64- or 256-channel scanners (Brilliance, the IQon, and the iCT, Philips Healthcare, Best, The Netherlands). The imaging parameters were as follows: tube voltage of 120 kV; effective tube current of 250 mAs; collimation of 64 × 0.625; pitch of 0.39; rotation time of 0.5; raw slice thickness/increment of 0.9/0.45 mm; and axial reconstruction slice thickness/increment of 5/5 mm. The scan range extended from the base of the skull to the vertex. Stroke MR images were taken at 3 Tesla scanners (Achieva or Ingenia, Philips Healthcare, Best, The Netherlands) with 8-channel or 32-channel head coils, using the following image parameters; for DWI, repetition time = 3,000 ms, echo time = 72 ms, flip angle = 90°, number of excitation = 2, a field of view = 230 × 230 mm, matrix size = 160 × 160, and slice thickness = 5 mm; for FLAIR, repetition time = 11,000 ms, echo time = 125 ms, flip angle = 90°, number of excitation = 1, a field of view = 182 × 230 mm, matrix size = 352 × 263 mm, and slice thickness = 5 mm.

### 2.4. Development of reference standards and quantification of the diffusion-FLAIR mismatch

NCCT images were initially pre-processed by skull stripping, cropping, and intensity normalization. The DWI and FLAIR images were skull-stripped and automatically co-registered onto the pre-processed NCCT images using rigid body transformation (the SimpleITK packages in Python) (19, 20). The NCCT, DWI, and FLAIR images were, therefore, spatially aligned in the same space. An experienced neuroradiologist visually inspected the registration results and made manual adjustments using the ITK-SNAP (version 3.8^1^) when the co-registration was suboptimal (21). Board-certified vascular neurologists (BJK, NS), a neuroradiologist (RM), and interventional neuroradiologists (FB and PC) manually segmented high signal lesions on every slice on DWI and FLAIR images referring to ADC images using the ITK-SNAP.

DWI-FLAIR mismatch was defined as a region where DWI revealed a high signal intensity implying ischemic injury but no or only slight changes on the FLAIR image, indicating potentially minute irreversible infarction (22). Raters were recommended to compare the area of high signal intensity on the DWI with the corresponding area of low signal intensity on the apparent diffusion coefficient (ADC) map. However, no specific ADC threshold was given, considering the relatively greater ADC value in the ischemic penumbra (23). In evaluating FLAIR images, a high signal intensity of less than 15% greater than the contralateral intact region was not considered indicative of a FLAIR lesion (24). When the FLAIR high signal intensity lesion occupied more than one-third of the corresponding DWI lesion, the DWI-FLAIR mismatch was not counted.

After achieving a consensus on the DWI-FLAIR mismatch with a random sample of 50 image sets, three raters independently evaluated the remainder. Disputes between raters were settled by majority vote. The interclass correlation coefficient for DWI-FLAIR mismatch on MR images was 0.94 [95% CI, 0.73–1.00]. Six months following the segmentation of the lesion, DWI-FLAIR mismatch was rated based on the presence or absence of the mismatch.

### 2.5. Deep learning model

**Figure 2:**
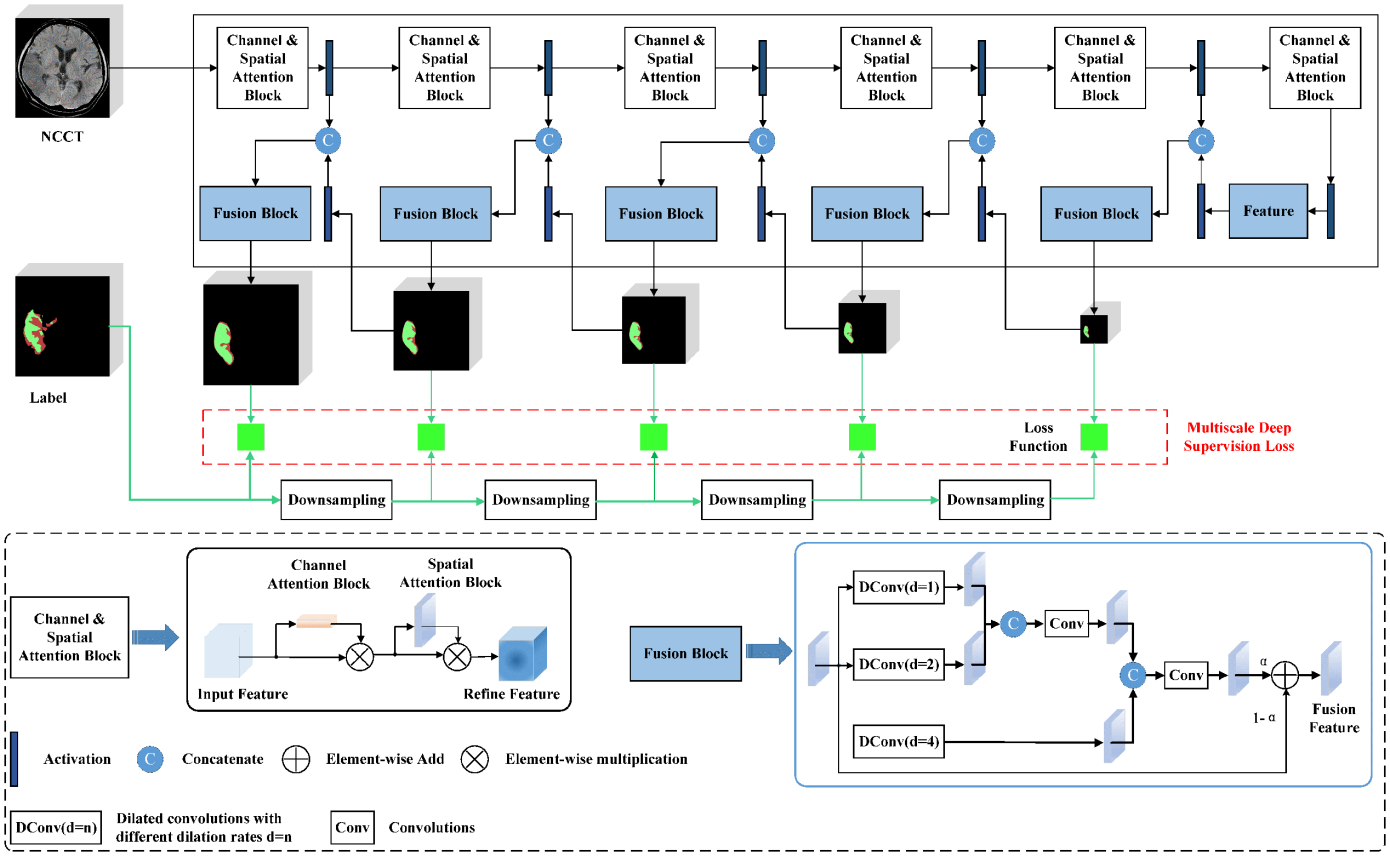
The proposed full-resolution deep learning architecture.

An automated lesion segmentation framework was built based on the nnU-net architecture (25, 26). The proposed DL model employed a full-resolution training strategy with a deep-supervision mechanism. Specifically, a channel and spatial attention block were designed to enable the model to focus on the salient areas of images at different scales and acquire compact and conducive features (27). To fully utilize multi-scale context information, a scale-aware pyramid fusion module with three parallel dilated convolutions with varying dilation rates of 1, 2, and 4 was employed and fused the information at different scales (Figure 2) (28).

To train and choose the optimum model, a five-fold cross-validation was executed. Spatial augmentations, including rotation, scaling, and low-resolution simulation, were applied in three dimensions to boost the diversity of training data. Dice and cross-entropy loss functions were utilized to supervise the learning (29).

### 2.6. Evaluation metrics

The proposed segmentation method was quantitatively evaluated using a spatial overlap metric of the Dice Coefficient (DC), and two boundary distance error metrics, average symmetric surface distance (ASSD) and 95th percentile of the Hausdorff Distance (HD95), were compared to the reference standard of manual segmentation. DC is a spatial overlap index ranging from 0 to 1; 1 indicates a perfect overlap between the reference standard and predicted segmentation and 0 indicates no overlap. The ASSD computes the average difference between the segmented object’s surface and the reference in three-dimensional space. The HD95 represents the greatest Hausdorff distance and ignores the top 5% with the biggest surface distance error. For ASSD and HD95, 0 mm indicates a perfect segmentation. Depending on the distribution of the dataset, Pearson and Spearman’s correlations were used to compare the segmented lesion volumes. Interclass coefficients were calculated to estimate the interobserver agreement of the DWI-FLAIR mismatch rating. A confusion matrix was created to assess the classification performance, from which the sensitivity, specificity, accuracy, and AUC-ROC score were derived. All of these metrics were computed in 3D space at the level of the patient.

### 2.7. Application of the developed model to the practice

After developing the image prediction model, we tested the performance and practicality of the model in a quasi-practice setting. Vascular neurologists with more than 10 years of practice (BJK) and vascular fellows in training (YSK and JWK) evaluated the presence of DWI-FLAIR mismatch from NCCT images without clinical information or the model’s prediction (evaluation round #1). Two weeks later, they re-evaluated NCCT images while the model’s prediction outputs were provided (evaluation round #2). Human raters were blinded to clinical information, time from onset, location of ischemic lesion, and the presence of the DWI-FLAIR mismatch. A discrepancy was settled by a majority vote, and the consensus ratings were evaluated against the prediction model’s output.

### 2.8. Statistical analysis

Baseline characteristics of study participants were summarized as means ± standard deviations, medians [interquartile ranges], and frequencies (percentages), as appropriate. Distributions of variables were compared using *t*-tests for interval variables and chi-squared tests for categorical variables. Using the bootstrap method for the area-under-the-curve of receiver-operating-characteristics (AUC-ROC) analysis, the AI model’s performance was tested (30). The statistical significance thresholds were set at a two-tailed *p*-value < 0.05. Statistical analyses were performed using R version 4.1.2 (R Foundation for Statistical Computing).

## 3. Results

### 3.1. Patient characteristics

Of 453 acute ischemic stroke patients who had taken NCCT – DWI – FLAIR images within 1 h of one another during the 10-year study period, 222 (49%) cases met the prespecified selection criteria for the current study. The mean age of the study population was 71.8 ± 12.8 years, and 123 (55%) of them were male. The baseline NIHSS score at arrival was a median of 11 [IQR, 6–18]. 149 (67%) received acute stroke treatment [120 (54%) intravenous thrombolysis and 77 (35%) endovascular recanalization]. Baseline characteristics were comparable between the derivation and validation cohort except that atrial fibrillation was more prevalent, and blood pressure and glucose levels were higher in the validation set (Table 1).

**Table 1:** Clinical characteristics of study subjects.

Variables	All patients (*n*, 222)	Development set (*n*, 130)	Test set (*n*, 92)	P-for-difference
Male sex	123 (55%)	73 (56%)	50 (54%)	0.90
Age	71.8 ± 12.8	71.1 ± 13.0	70.3 ± 12.7	0.68
Baseline NIHSS score	11 [6–18]	11 [6–18]	11 [6–16]	0.45
LKW to arrival (minutes)	109 [57–291]	101 [54–251]	119 [60–315]	0.47
Pre-stroke functional dependency (mRS ≥1)	27 (12%)	17 (14%)	10 (11%)	0.77
Stroke mechanisms				0.14
Large artery atherosclerosis	57 (26%)	37 (29%)	20 (22%)	
Small vessel occlusion	3 (1%)	0	3 (3%)	
Cardioembolism	122 (55%)	67 (52%)	55 (60%)	
Other determined etiology	14 (6%)	9 (7%)	5 (6%)	
Undetermined etiology	25 (11%)	17 (13%)	8 (9%)	
Hypertension	160 (72%)	93 (72%)	67 (73%)	0.95
Diabetes	67 (30%)	36 (28%)	31 (34%)	0.42
Dyslipidemia	58 (26%)	35 (27%)	23 (25%)	0.87
Habitual smoking	70 (32%)	44 (34%)	26 (28%)	0.46
Atrial fibrillation	99 (45%)	49 (38%)	50 (54%)	0.02
Occlusion location				0.32
Internal carotid artery	16 (7%)	9 (7%)	7 (8%)	
Middle cerebral artery	149 (67%)	83 (64%)	66 (72%)	
Anterior cerebral artery	5 (2%)	5 (4%)	0	
Posterior cerebral artery	11 (5%)	9 (7%)	2 (2%)	
Vertebrobasilar artery	13 (6%)	6 (5%)	7 (8%)	
Multiple occlusion	28 (12%)	18 (14%)	10 (10%)	
Recanalization treatment	149 (67%)	89 (69%)	60 (65%)	0.72
Intravenous thrombolysis	144 (65%)	87 (67%)	57 (62%)	0.53
Endovascular therapy	78 (35%)	43 (33%)	35 (38%)	0.53
Hemoglobin (mg/dL)	13.5 ± 2.2	13.4 ± 2.4	13.5 ± 1.9	0.78
Blood urea nitrogen (mg/dL)	18 ± 8	18 ± 10	17 ± 6	0.36
Creatinine (mg/dL)	0.9 ± 0.4	1.0 ± 0.5	0.9 ± 0.4	0.57
Total cholesterol (mg/dL)	162 ± 41	161 ± 39	163 ± 42	0.67
Low-density lipoprotein (mg/dL)	95 ± 33	95 ± 31	95 ± 36	0.88
Hemoglobin A1c (%)	6.1 ± 1.1	6.0 ± 1.0	6.3 ± 1.3	0.12
Blood glucose on arrival (mg/dL)	139 ± 57	132 ± 44	148 ± 71	0.03
Systolic blood pressure (mm Hg)	155 ± 26	150 ± 25	162 ± 27	<0.01
Diastolic blood pressure	83 ± 17	81 ± 16	87 ± 17	0.01
NIHSS score at discharge	5 [2–11]	5 [3–10]	5 [2–11]	0.56
Early neurological deterioration	39 (18%)	18 (14%)	21 (23%)	0.12
mRS score 0–1 at 3 months	70 (34%)	46 (38%)	24 (29%)	0.21

All stroke images were taken in the order of NCCT – DWI – FLAIR sequences. The image scan was commenced after a median of 139 [81–326] min after the time last known well (LKW); the median time from the first NCCT and the last FLAIR image was 39 [32–50] min (Table 2). The time indices were similar between derivation and validation sets. The DWI-FLAIR mismatch was detected in 43 patients (47%).

**Table 2:** Lesion volume and image acquisition metrics.

Variables	All patients (*n*, 222)	Development set (*n*, 130)	Test set (*n*, 92)	P-for-difference
Lesion volume on DWI (mL)	11.0 [4.4–35.7]	11.8 [4.4–35.0]	10.6 [4.4–36.3]	0.66
Lesion volume on FLAIR (mL)	1.9 [0.5–7.0]	2.1 [1.0–8.1]	1.1 [0–5.0]	<0.01
LKW to CT (minute)	139 [81–326]	128 [76–317]	146 [90–330]	0.86
LKW to DWI (minute)	170 [111–351]	164 [107–334]	175 [118–353]	0.83
LKW to FLAIR (minute)	183 [125–362]	175 [119–343]	188 [129–366]	0.66
CT to DWI (minute)	29 [24–35]	30 [23–35]	29 [25–34]	0.92
CT to FLAIR (minute)	39 [32–50]	40 [31–50]	39 [34–50]	0.69
DWI to FLAIR (minute)	9 [5–17]	10 [5–17]	7 [5–18]	0.63

*DWI, diffusion-weighted image; FLAIR; fluid-attenuated inversion recovery; CT, computed tomography; LKW, last known well.

### 3.2. Automated segmentation of DWI and FLAIR lesions

**Figure 3:**
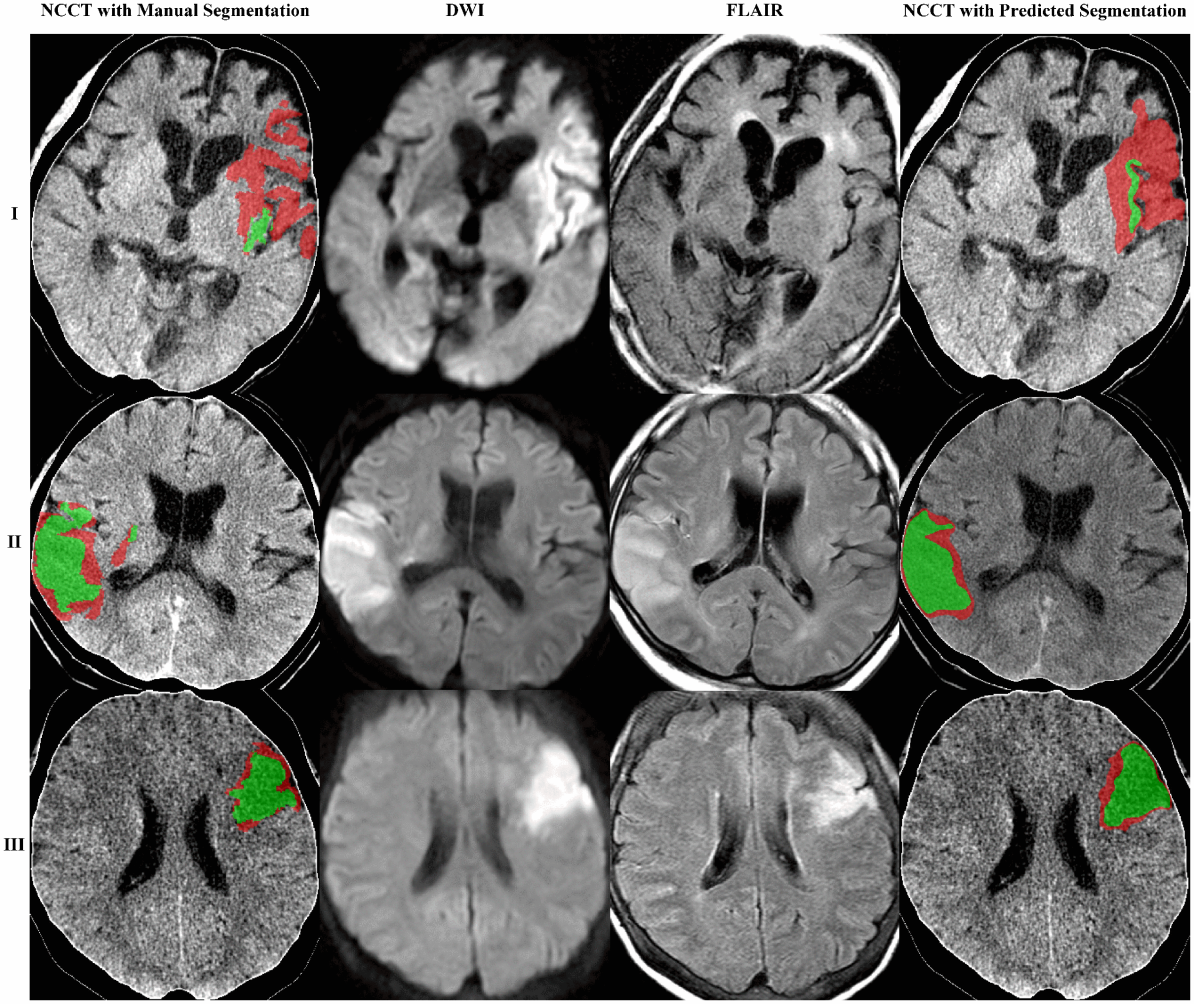
The proposed model obtained three segmentation examples. The images are shown as NCCT overlaid with the manually or algorithm-segmented DWI and FLAIR lesions colored in red and green, respectively. Case example of mismatch (I) and no mismatch (II and III).

A deep-learning model to segment ischemic lesions on the DWI and FLAIR images was initially developed. When the derived deep learning model was applied to the 92 patients in the test set, the DCs were a mean of 39.1% (95% CI, 31.4–46.8) for DWI lesions and 18.9% (95% CI, 12.3–25.6) for FLAIR lesions; the ASSDs were 9.1 mm (95% CI, 5.0–13.1) for DWI lesions and 10.6 mm (95% CI, 6.3–14.8) for FLAIR lesions; the HD95 was 29.3 mm (95% CI, 22.0–36.6) for DWI and 29.3 mm (22.5–36.1) for FLAIR lesions. The differences between predicted and reference area volumes were − 11.4 mL (95% CI, −17.3 to −5.4) for DWI and −4.5 mL (95% CI, −6.6 to −2.4) for FLAIR lesions. Considering the low spatial resolution and delayed appearance of ischemia on NCCT images, image sets with their DWI lesion ≥15 mL were selected, and the DL model’s performance was re-evaluated. In general, the model output showed a greater similarity to DWI and FLAIR lesions in the larger ischemic lesion subgroup (Table 3; Figure 3).

**Table 3:** The prediction ability of DWI and FLAIR lesions by a deep learning model in the test cohort (92 cases).

	DWI lesion volume < 15 mL		DWI lesion volume ≥ 15 mL		All samples	
	DWI	FLAIR	DWI	FLAIR	DWI	FLAIR
Lesion volume (ground truth; mL)	7.27 (5.44–9.11)	2.56 (1.16–3.96)	65.37 (43.61–87.13)	13.33 (8.03–18.62)	36.13 (23.12–49.14)	7.62 (4.61–10.62)
Prediction volume (mL)	8.53 (−0.29 to 17.35)	0.5 (0.0–0.99)	45.07 (25.73–64.42)	6.0 (2.92–9.09)	24.78 (13.79–35.77)	3.16 (1.47–4.84)
$\delta V_{\mathrm{diff}}\;$ (mL)	1.26 (−6.67 to 9.19)	−2.06 (−3.21 to −0.91)	−20.3 (−30.81 to −9.78)	−7.32 (−11.27 to −3.37)	−11.35 (−17.26 to −5.43)	−4.46 (−6.56 to −2.36)
$\left|\delta V_{\mathrm{diff}}\right|\;$ (mL)	8.53 (2.25–14.81)	2.08 (0.94–3.22)	26.77 (18.26–35.27)	8.56 (4.93–12.18)	16.82 (11.75–21.89)	5.09 (3.08–7.1)
Volume correlation	0.6 (0.38–0.81)	0.42 (0.18–0.65)	0.84 (0.78–0.9)	0.67 (0.58–0.76)	0.76 (0.68–0.84)	0.61 (0.52–0.7)

Data are the mean values with the 95% confidence interval in parentheses.

DWI, diffusion-weighted image; FLAIR, fluid-attenuated inversion recovery; $\delta V_{\mathrm{diff}}$ , difference in volume; $\left|\delta V_{\mathrm{diff}}\right|$ , absolute difference in volume.

### 3.3. Pixelwise prediction models for the DWI-FLAIR mismatch on NCCT

**Figure 4:**
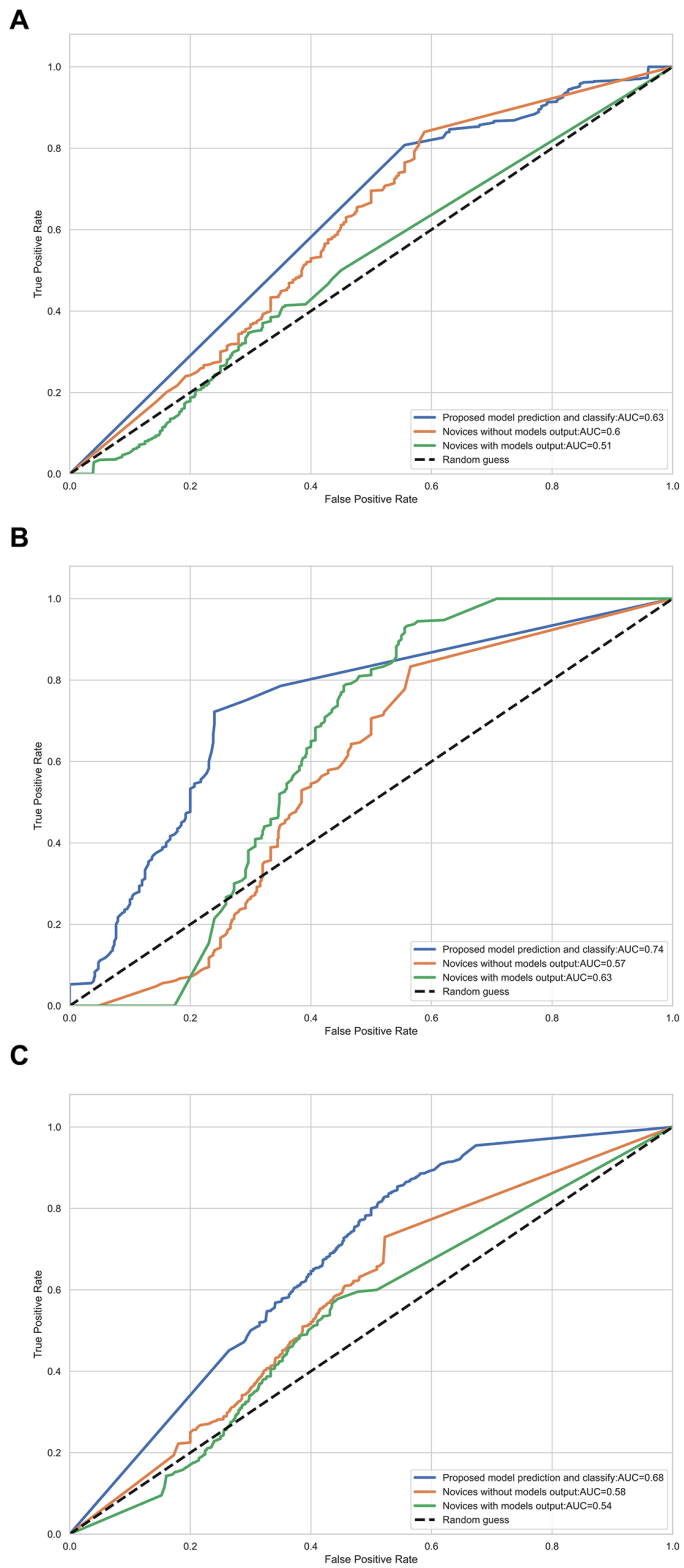
Receiver-operator characteristic (ROC) curves for each radiologist using DWI and FLAIR images and NCCT with or without the model’s prediction for detecting DWI/FLAIR mismatch. **(A)** DWI volume range (2, 15) mL; **(B)** DWI volume range ≥15 mL **(C)** All data.

An expert panel of vascular neurologists and neuroradiologists judged the presence of DWI-FLAIR mismatch from the NCCT images, yielding moderate prediction with an accuracy of 0.63 and an AUC-ROC of 0.635 [95% CI, 0.554–0.713]. Experts’ evaluations were similar by the size of the DWI lesion; AUC-ROC of 0.586 for DWI lesions between 2–15 mL and 0.596 for DWI lesions ≥15 mL. A novice panel with only 1 or 2 years of clinical vascular neurology fellowship training evaluated the NCCT images and showed relatively mediocre predictability even for image sets from DWI lesions ≥15 mL. However, when provided with the DL model’s output, the novice panel’s evaluation of DWI-FLAIR mismatch from NCCT images improved in accuracy (from 0.537 to 0.610) and AUC-ROC (from 0.493 to 0.613) in the larger lesion subgroup (Table 4; Figure 4).

**Table 4:** Human raters’ estimation of the DWI-FLAIR mismatch on NCCT images with or without the deep learning model’s output.

		Precision	Recall	Accuracy	Specificity	Sensitivity	AUC-ROC [95% CI]	P-for-difference of AUC-ROC’s
**All image sets**								
Experts	Without model’s output	0.585	0.692	0.630	0.551	0.721	0.635 [0.554–0.713]	<0.01
Novices	Without model’s output	0.528	0.571	0.554	0.653	0.442	0.547 [0.466–0.629]	Reference
Novices	With model’s output	0.500	0.548	0.533	0.694	0.349	0.522 [0.441–0.601]	<0.01
**DWI lesions, 2–15 mL**								
Experts	Without model’s output	0.578	0.833	0.608	0.208	0.963	0.586 [0.515–0.664]	0.01
Novices	Without model’s output	0.609	0.536	0.569	0.625	0.519	0.567 [0.451–0.674]	reference
Novices	With model’s output	0.500	0.463	0.471	0.792	0.185	0.489 [0.394–0.581]	0.01
**DWI lesions >15 mL**								
Experts	Without model’s output	0.625	0.667	0.659	0.880	0.313	0.596 [0.490–0.701]	<0.01
Novices	Without model’s output	0.385	0.607	0.537	0.680	0.313	0.493 [0.368–0.615]	reference
Novices	With model’s output	0.500	0.714	0.610	0.600	0.625	0.613 [0.481–0.745]	<0.01

DWI, diffusion-weighted image; FLAIR, fluid-attenuated inversion recovery; AUC-ROC, area-under-curve of receiver-operating characteristics.

## 4. Discussion

Through 222 NCCT – DWI – FLAIR image sets of acute ischemic stroke patients acquired within 1 h of one another, we constructed a deep learning model predicting DWI-FLAIR lesions and applied these to the NCCT images. The predictability of the AI model remained modest, with increased specificity in a larger infarct group with a DWI lesion volume greater than 15 mL. The model was demonstrated to support the interpretation of stroke physicians with limited clinical expertise in the large infarction group of ≥15 mL.

The purpose of acute stroke imaging is, among other things, to distinguish viable brain tissue from irreparable infarctions to maximize the benefit of recanalization treatment and minimize the risk of hemorrhagic complications (31). MR scans are generally thought to demonstrate great accuracy in characterizing ischemic injuries. The signal change on FLAIR has been attributed to vasogenic edema following the initial cytotoxic edema visualized on DWI. The DWI-FLAIR mismatch, a high signal lesion on DWI in contrast to a normal or minimally increased signal intensity on FLAIR, may imply potentially reversible ischemia. However, it is not entirely accepted in clinical practice yet. The safety and efficacy of intravenous thrombolysis based on the DWI-FLAIR mismatch were documented in randomized clinical trials (11, 12). A single-center study showed the utility of the DWI-FLAIR mismatch as a case selection for endovascular recanalization treatment (10). Therefore, DWI-FLAIR mismatch may be considered a tissue clock, beyond the role of a time clock, that may guide the reperfusion treatment.

However, the diffusion-FLAIR mismatch is not generally utilized in real-world clinical decisions due to (1) the limited accessibility of MR scanners for acute stroke care and (2) the ambiguity in determining ischemic signals on FLAIR images. Our study aims to develop a practical AI model to aid hyperacute stroke treatment decisions in rural hospitals without MR machines. Our model’s output, solely based on NCCT images, showed a reasonable correlation to DWI and FLAIR lesions (correlation coefficient, 0.84 and 0.67) in a group of DWI lesions greater than 15 mL.

The goal of current AI models for medical image interpretation is to provide a comparable reading to human experts (32). The DWI-FLAIR mismatch, by definition, requires MR scans to be determined. Recognition of the mismatch in NCCT images may not be feasible to the naked human eye due to its limited spatial resolution and discrimination of radiodensity signals (33). We tried to sidestep the technical limitation by constructing separate models for DWI and FLAIR lesions and summed together those outputs to make a final decision. The developed deep learning technique made use of the sophisticated convolutional neural network architecture while applying the attention mechanism and multi-resolution supervision strategy (25). Our model demonstrated that the proposed strategy was able to pick up the weak mismatch signals on NCCT, notably for the lesions ≥15 mL.

Although the DWI-FLAIR mismatch is not commonly used in clinical practice, the “tissue clock” visualized on NCCT may facilitate intravenous thrombolysis in rural settings where MR scanners, experienced staff, and timely transfer are not always accessible. We provided the output of our AI model from NCCT to vascular neurology fellows with only 1 or 2 years of clinical practice experience. Their judgment of a larger DWI-FLAIR mismatch on NCCT significantly improved with aid from the AI model, from an AUC-ROC curve of 0.49 without output to 0.61 with output. The greater discrimination values imply our model’s feasibility and applicability in general clinical practice with restricted resource conditions.

This study has several limitations. The predictability of our model in terms of lesion volume and Dice coefficient is modest. Developing a deep learning model with greater accuracy requires large data. However, the NCCT – DWI – FLAIR image sets acquired within 1 h are scarce in real-world clinical practice, as only 5% of more than 8,000 acute stroke patients produced such sets. Our study was based on single-center data without an external cohort to validate. We excluded cases with tiny DWI lesions of less than 2 mL, considering the limited spatial resolution of NCCT images. We supposed no substantial pathologic changes during the 1-h image acquisition period from NCCT to FLAIR images. Intravenous alteplase was given in 65% of cases, but no endovascular treatment was attempted during the 1 h.

## 5. Conclusion

We developed a deep learning model predicting the DWI-FLAIR mismatch on NCCT images in 222 acute ischemic stroke patients who acquired all the images within a 1-h period. The AI model modestly predicted both DWI and FLAIR lesions on NCCT images, and the output enhanced inexperienced readers’ interpretation. We were able to demonstrate that the AI model discerned minute ischemic changes in the NCCT image. Our study may suggest that deep learning technology may improve acute stroke care and critical decision-making on the intravenous thrombolysis for ischemic stroke patients with substantial ischemic lesions.

## Data availability statement

The raw data supporting the conclusions of this article will be made available by the authors, without undue reservation.

## Ethics statement

The studies involving human participants were reviewed and approved by the institutional review board of the Seoul National University Bundang Hospital, they approved the data analysis, image evaluation, and modeling process (B-2102/667-106). The patients/participants provided their written informed consent to participate in this study.

## Author contributions

BK collected patients’ data, including images, evaluated the image, wrote the initial draft, and managed research funding. KZ analyzed the data and managed research funding. KZ and WQ developed the nnU-net model. NS, RM, PC, FB, JK, and YK evaluated the image. H-JB collected patients’ data. BM conceived the study idea, analyzed the data, made critical intellectual contributions to the manuscript, and led the research team. All authors contributed to the article and approved the submitted version.

## Funding

The current study was supported by the intramural research fund from Seoul National University Bundang Hospital [14-2016-0017], endowed to BK. This research was supported by a grant of the Korea Health Technology R&D Project through the Korea Health Industry Development Institute (KHIDI), funded by the Ministry of Health and Welfare, Republic of Korea (grant number: HI22C0454), endowed to BK. This research was funded by Natural Science Basic Research Plan in Shaanxi Province of China (Program No. 2021JM-413), endowed to KZ.

## Conflict of interest

The authors declare that the research was conducted in the absence of any commercial or financial relationships that could be construed as a potential conflict of interest.

## Publisher’s note

All claims expressed in this article are solely those of the authors and do not necessarily represent those of their affiliated organizations, or those of the publisher, the editors and the reviewers. Any product that may be evaluated in this article, or claim that may be made by its manufacturer, is not guaranteed or endorsed by the publisher.

## Footnotes

^1^http://www.itksnap.org/
